# Less is more: Aesthetic liking is inversely related to metabolic expense by the visual system

**DOI:** 10.1093/pnasnexus/pgaf347

**Published:** 2025-12-02

**Authors:** Yikai Tang, William A Cunningham, Dirk B Walther

**Affiliations:** Department of Psychology, University of Toronto, 100 St. George Street, Toronto, ON, Canada M5S 3G3; Vector Institute, 101 College Street, Toronto, ON, Canada M5G 1L7; Department of Psychology, University of Toronto, 100 St. George Street, Toronto, ON, Canada M5S 3G3; Vector Institute, 101 College Street, Toronto, ON, Canada M5G 1L7; Department of Computer Science, University of Toronto, 40 St. George Street, Toronto, ON, Canada M5S 2E4; Schwartz Reisman Institute for Technology and Society, University of Toronto, 101 College Street, Toronto, ON, Canada M5G 1L7; Department of Psychology, University of Toronto, 100 St. George Street, Toronto, ON, Canada M5S 3G3

**Keywords:** aesthetics, metabolic activity, visual cortex, BOLD, convolutional neural network

## Abstract

Energy efficiency is a major driving force in the evolution of organisms, and previous research implies that humans may have evolved pleasure-based signals to guide optimal actions. But could this energy-saving heuristic also apply to aesthetic pleasure? We test this hypothesis using both an in silico model of the visual system (VGG19) and human observers, finding strong evidence in both. First, we measure the proxy for metabolic cost incurred by VGG19—either pretrained for object and scene categorization or randomly initialized—as it processes 4,914 images of objects and scenes, revealing an inverse relationship between aesthetic preferences and metabolic cost, and only in the pretrained model. Next, we compare aesthetic ratings of visual stimuli to metabolic activity in the human visual system, measured via the blood oxygen level-dependent signal during functional magnetic resonance imaging. We observe the same inverse relationship between blood oxygen level dependent signals and aesthetic preferences in both early visual regions (V1, V2, and V4) and higher-level regions (fusiform face area, occipital place area, and parahippocampal place area). These findings suggest that aesthetic preferences may at least partially arise from an affective heuristic favoring low-energy states, and they offer a unified framework linking empirical evidence on visual discomfort with theories of processing fluency, image complexity, and prototypicality, providing a straightforward model for understanding aesthetic judgments.

Significance Statement How do our brain's adaptive processes shape our aesthetic appreciation? We propose that visual aesthetic appreciation may be a manifestation of an energy-conserving affect heuristic: aesthetic pleasure from viewing an image is related to the amount of energy the visual system uses to process it. Our findings reveal an inverse relationship between viewing pleasure and the processing metabolic cost, demonstrated both in artificial neural networks and the human brain. This research marks the first direct evidence linking visual aesthetics to energy consumption in the visual system, connecting aesthetic appreciation to adaptive brain mechanisms. Our work offers an intuitive, biologically grounded framework for understanding various factors influencing aesthetic experience.

## Introduction

Energy efficiency is a fundamental challenge for all organisms, influencing both physical and cognitive activities. To survive, organisms must interact with their environment adaptively and efficiently. In biological systems, this often involves balancing the metabolic costs of actions—whether physical or cognitive—with the rewards those actions yield (1). Just as it is metabolically inefficient to expend excessive physical effort, such as jumping unnecessarily high for food, cognitive operations can also be inefficient and costly. Despite the importance of this cost–benefit balance, directly calculating these ratios is often impractical. One proposed solution is that organisms may rely on affective heuristics, using pleasure-based signals to guide their preferences toward optimal actions (2–5). If these heuristics are embedded throughout neural architectures, a preference for low-energy states should manifest in primary perceptual experience, such as aesthetic preferences—pleasure derived from interacting with the external environment.

Organisms may employ strategies, whether learned or hardwired, to select environments that meet certain needs. We may have developed hedonic signaling as a simple and rapid readout for this purpose, potentially having a downstream effect on people's sense of preference. Environmental features that signal adaptive value can increase the perceived pleasure of images (6–8), while features indicating potential harm, such as sharp contours (9, 10) or the potential for concealed threats (6), tend to elicit dislike. In these cases, the perceived quality (e.g. safety) has direct survival benefits. In this study, we investigated a more fundamental quality: the metabolic costs associated with processing and representing external stimuli.

Beyond adaptive challenges, managing the costs of processing the external world is a more universal and critical concern. Energy expenditure, in particular, constrains our visual processing. The brain consumes 20% of the body's energy (11), and the visual system alone accounts for about 44% of the brain's energy consumption (12). Affect may serve as a signal for these costs, as suggested by prior evidence. Studies on processing fluency have shown that fluent perceptual processing leads to positive affective responses (13, 14). Some research also suggests that metabolic efficiency plays a role in basic visual processing. Studies examining neural responses using limited image datasets or low-resolution images have shown that sensitivity to visual discomfort is positively related to the intensity of neuronal excitation (15–19). We propose that the metabolic efficiency of the visual system during encoding is rewarding and manifests as aesthetic pleasure.

This proposal aligns with Berlyne's two-factor model (20), which posits that aesthetic appreciation may be shaped by a tedium factor and a positive-habituation factor, with preference increasing alongside positive-habituation but decreasing with increased tedium. This model explains why overly simple stimuli, such as a blank white room, fail to induce aesthetic pleasure despite requiring minimal cognitive effort—they are tedious and elicit minimal arousal. In this study, we do not aim to examine both components of the model. Instead, we focus on one—the positive-habituation component—pursuing a direction consistent with it by investigating the physiological factor of metabolic costs. In this vein, previous research supporting this model has largely focused on artificial or simplistic visual stimuli, such as dots and stripes (14–16, 19) or simple geometric shapes (21). However, comprehensive studies involving complex, real-world images are lacking, and physiological measures of positive-habituation in these contexts remain underexplored.

**Fig. 1.**
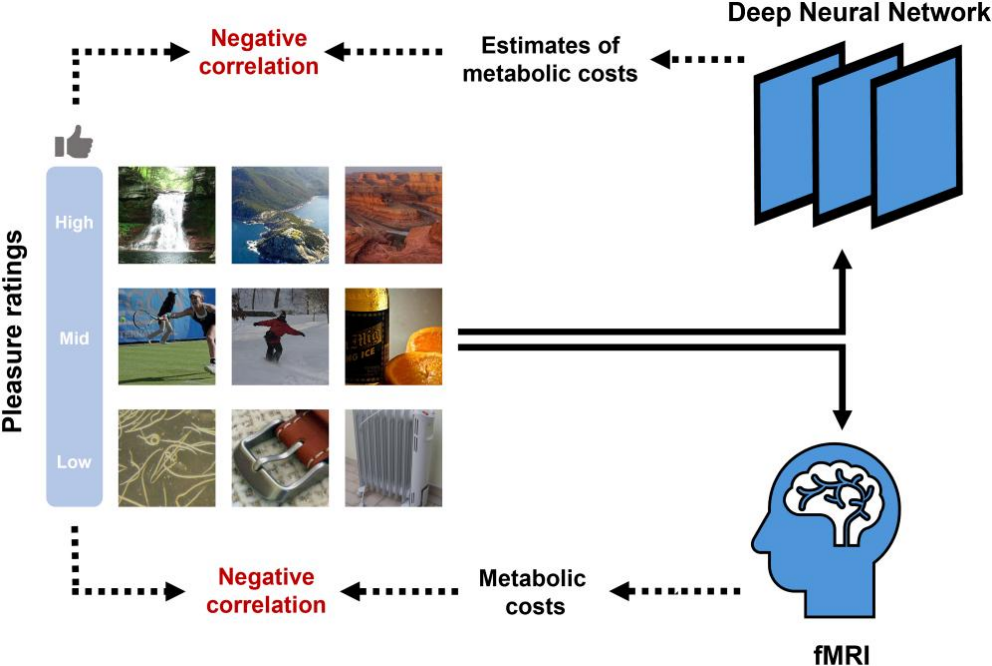
Conceptual roadmap of the present study. To examine the relationship between the metabolic costs of visual processing and aesthetic pleasure, we used both computational and physiological measures to quantify metabolic costs during visual processing: (1) Model-derived estimates of metabolic costs based on the activation of a deep neural network; (2) Metabolic activity of human brains, specifically in the visual processing areas. We found that both measures were inversely related to aesthetic pleasure. Example stimuli are reproduced from the BOLD5000 dataset (24).

We hypothesize that aesthetic preferences for visual stimuli are inversely related to the metabolic cost of their neural representations within the visual system. To test this hypothesis, we used a dual approach combining computational modeling and human data validation with a dataset of nearly 5,000 images (Fig. 1). First, we employed an artificial neural network, VGG19 (22), which has been shown to parallel human visual processing (23), to estimate the metabolic costs associated with encoding real-world images. This analysis aimed to provide a process-pure demonstration of the hypothesized relationship, showing that it exists even in basic feedforward processes. Second, we assessed the metabolic activity of the human visual system using functional magnetic resonance imaging (fMRI) while participants viewed the same images. In both cases, we found strong evidence supporting the hypothesized inverse relationship between metabolic expense and aesthetic liking (Fig. 1). These findings provide the first large-scale, direct evidence of a physiological basis for visual aesthetics rooted in the metabolic costs of neural processing.

## Results

### Image dataset and data collection

We used the BOLD5000 image dataset (24) to collect both human aesthetic ratings and neural network activation measures. The dataset covers a wide range of real-world images from three image sets, including 1,999 object images from COCO (25), 1,915 scene and object images from (26), and 1,000 images from Scene Images (with scene categories based on categories in Scene UNderstanding database) (27). The diversity of the images in the dataset contributes to the ecological validity of our investigation of aesthetic perception, making it less constrained to particular categories of visual stimuli.

The aesthetic pleasure measures for all blood oxygen level dependent (BOLD) 5,000 images were obtained by Ref. (28) in a population of 1,118 participants recruited using Amazon Mechanical Turk from Canada and the United States, each evaluating the images’ enjoyment on a 5-point scale. The collected ratings (on average 50 ratings per image) were then transformed into z-scores separately for each participant. For each image, the normalized ratings were averaged over participants to obtain the final measures used in our study.

### Proxy of metabolic costs in deep neural networks

To examine whether there is a relationship between the metabolic costs of visual representations and aesthetic pleasure, we first used a deep convolutional neural network (DCNN) to obtain a model-based estimate of metabolic costs. The model was selected for its ability to approximate computational features of the human visual system (29–32). We selected the VGG19 model, which was trained on object and scene categorization using ImageNet (22). We defined the total number of active model neurons (activation level >0) during the processing of an image as a proxy measure of metabolic costs. Each active neuron served as a proxy for a neuronal spike train. Spikes are significantly more energy-intensive than resting potentials (33–35). Specifically, we recorded the feature maps of each parameterized layer (after the rectifying linear unit [ReLU] activation function) in the network in response to each input image. Each nonzero element in the feature maps was counted as an active neuron. For comparison, we performed the same process for 1,000 randomly initialized VGG19 models to obtain a null distribution of networks with the same overall architecture that were not trained for categorization.

**Fig. 2.**
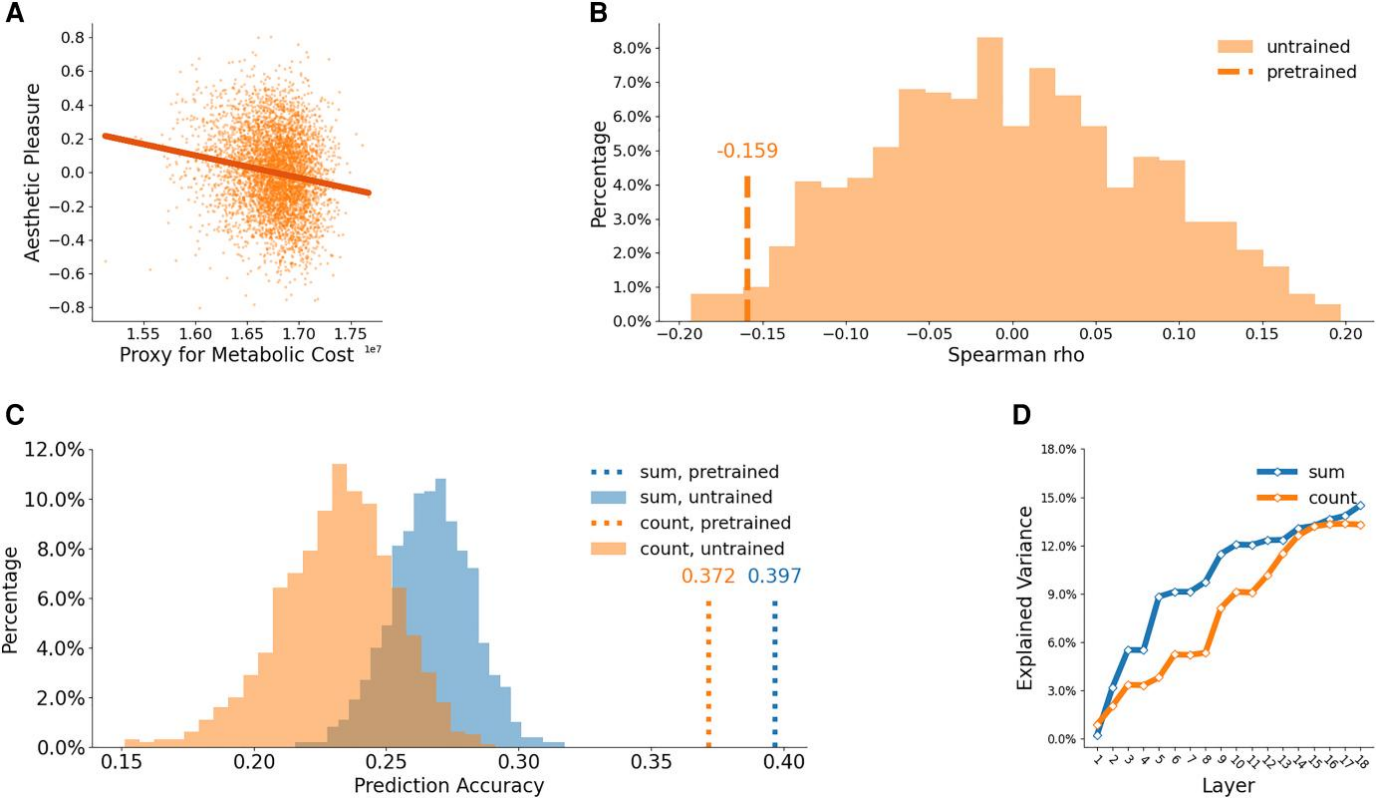
Relationship between aesthetic pleasure and model-derived estimates of metabolic costs in visual processing. A) Scatter plot of aesthetic pleasure over the number of active VGG19 units for all 4,914 images. B) Comparison between the correlation values of the pretrained model and the distribution of randomly initialized models (correlation between the count of active neurons and aesthetic pleasure). The dashed line shows the correlation for the pretrained model from (A). The linear combination of layer-wise metabolic costs accurately predicts aesthetic pleasure. C) The prediction accuracy of regression models fitted on untrained and pretrained VGG19 networks’ layer-wise metabolic costs, when metabolic costs were measured by counting the number of active neurons or summing up the total activations. D) Accumulation of explained variance in aesthetic pleasure by layer-wise costs, using either the total number of active neurons or the total activations as a conceptual proxy for metabolic costs. We evaluated the explanatory power of each layer's metabolic costs by controlling for the metabolic costs of the layers before it.

If the aesthetic pleasure signal is a manifestation of an energy-conservation affect heuristic, we should see an inverse relationship between the total number of active neurons in the representations of images in VGG19 and their pleasure ratings. We computed the image-by-image model-informed estimate of the metabolic cost of images as they were processed by VGG19 (the generic activation patterns are presented in the Supplementary material). We correlated these values with their aesthetic appeal according to ratings by human observers and found a significant negative correlation between the total number of active VGG19 neurons and the corresponding aesthetic appeal (Spearman rho = −0.16, *P* < 0.001; Fig. 2A). We compared this finding to 1,000 randomly initialized neural networks with the same architecture. Some of the untrained models (*n* = 396) also showed significant negative correlations. However, only 18 of them had a correlation that was lower than that of the VGG19 model trained for categorization (Fig. 2B). That is, the negative correlation of the trained model was significantly smaller compared to the null distribution at *P* = 0.018. This result suggests that the metabolic cost of representing the images is closely related to people's hedonic evaluation of the visual stimuli.

Our investigation of the correlation between the total number of active neurons and the pleasure ratings of the images imposed the assumption that the level of activity of each layer in the visual hierarchy should be equivalent in their contributions to the aesthetic hedonic signal. The assumption is a simplification of the situation in the visual system, where neural structures located at different levels of the visual hierarchy are likely to have different contributions to aesthetic judgment/perception. We thus took a step further to examine whether the combination of layer metrics with different weights can reveal a stronger relationship.

We fit a linear regression model with the number of active neurons of each layer as the predictor and aesthetic pleasure as the outcome variable, using 90% of the BOLD5000 dataset. We then tested the prediction accuracy of the model with the remaining 10%. We repeated the procedure, leaving out another 10% of the data in a cross-validation process that produced predictions for all images in the data set in turn. The model accounted for 13.3% of the variance in aesthetic pleasure and reached a prediction accuracy of 0.372 (Spearman correlation between predicted aesthetic pleasure and human ratings). Considering that aesthetic perception is a complex and multifactor cognitive phenomenon (14, 36, 37), the values indicate the crucial role of metabolic costs.

The prediction accuracies of the regression models fitted with the untrained networks’ activations were contained within the range from 0.151 to 0.291 (*M* = 0.231, SD = 0.022). That is, the correlation between the predicted and the measured aesthetic appeal of the regression model fitted on the data of the pretrained VGG19 is higher than any of those fitted upon the untrained models (*P* < 0.001; Fig. 2C). We also found that almost all layers in the network have an independent contribution to the aesthetic pleasure signal. When the perceptual information went from shallow to deep layers, the variance in aesthetic ratings explained by the layer-wise activations gradually increased from 0 to 13.30% (Fig. 2D). These observations are consistent with previous findings showing that subtle experiences of fluency at each level of visual processing may merge into a global feeling of fluency (38, 39), which lends further support to the role of metabolic costs in shaping aesthetic perception. We also conducted Spearman correlation tests for each layer (see Fig. S1) to examine the relationship of interest. Layers involved in higher-level visual processing, particularly the middle convolutional layers, generally showed negative correlations.

When considering the metabolic costs of visual information processing, it is possible that the total number of active neurons in the VGG-19 network may have obscured the individual intensity of each neuron's activation. We therefore measured the sum of the values of activities of all neurons to operationalize metabolic costs in an additional analysis. Remember that VGG-19 uses a rectifying linear unit (ReLU) as its nonlinearity. As a result, unit activity is either positive or clamped to zero. In line with our assumption, the regression model fitted with the sum of activity for each layer resulted in even higher prediction accuracy (Spearman rho = 0.397, *P* < 0.001; Fig. 2C). Again, none of the untrained models showed a higher prediction accuracy than the VGG19 network trained to categorize objects and scenes (from 0.216 to 0.317). And as before, each layer's total number of active neurons provides an independent contribution to predicting the variance in aesthetic pleasure (Fig. 2D). Regression models fitted on each image subset have relatively consistent prediction accuracy, with 0.387 for scenes, 0.475 for COCO, 0.421 for ImageNet (all *P* values <0.001).

To ensure these findings are robust to details in network architecture, we repeated the same analysis with Resnet50 (40), which is functionally even more similar to the human visual system (23). We found similar patterns of results. When a regression model was fitted for the relationship between layer-wise sum neural activities and aesthetic pleasure, it accounted for 9.5% of the variances in aesthetic pleasure and its predictions correlated with the ground truth ratings at a Spearman's rho of 0.313 (Fig. S2). The prediction accuracy of the pretrained model was larger than the values of all the untrained models (*P* < 0.001), the distribution of which ranged from 0.174 to 0.309 (*M* = 0.255, SD = 0.021).

### Metabolic costs in the human visual system

With deep neural network models, we found that the activation level of neurons in feedforward artificial neural networks is related to human-provided aesthetic preference. DCNNs are process-pure models of the fundamental proposed mechanisms. With DCNNs, we could examine the exact process of interest and isolate the theoretical components, namely the feedforward path, without potential confounds like feedback influence on the visual system from other systems. It should be noted that a DCNN is a simplification of the human biological visual system (29, 30). How do the results derived from DCNNs translate to the human visual system?

To test the relationship between metabolic cost and aesthetic appeal in the human visual system, we used the BOLD signal commonly used for fMRI. The BOLD signal measures the influx of fresh, oxygenated blood in response to the depletion of oxygen due to neural activity in a local region of the brain (41). Because oxygen is crucial for the metabolic process of aerobic respiration from which neurons acquire energy, the BOLD signal is a direct measure of metabolic energy expenditure.

**Fig. 3.**
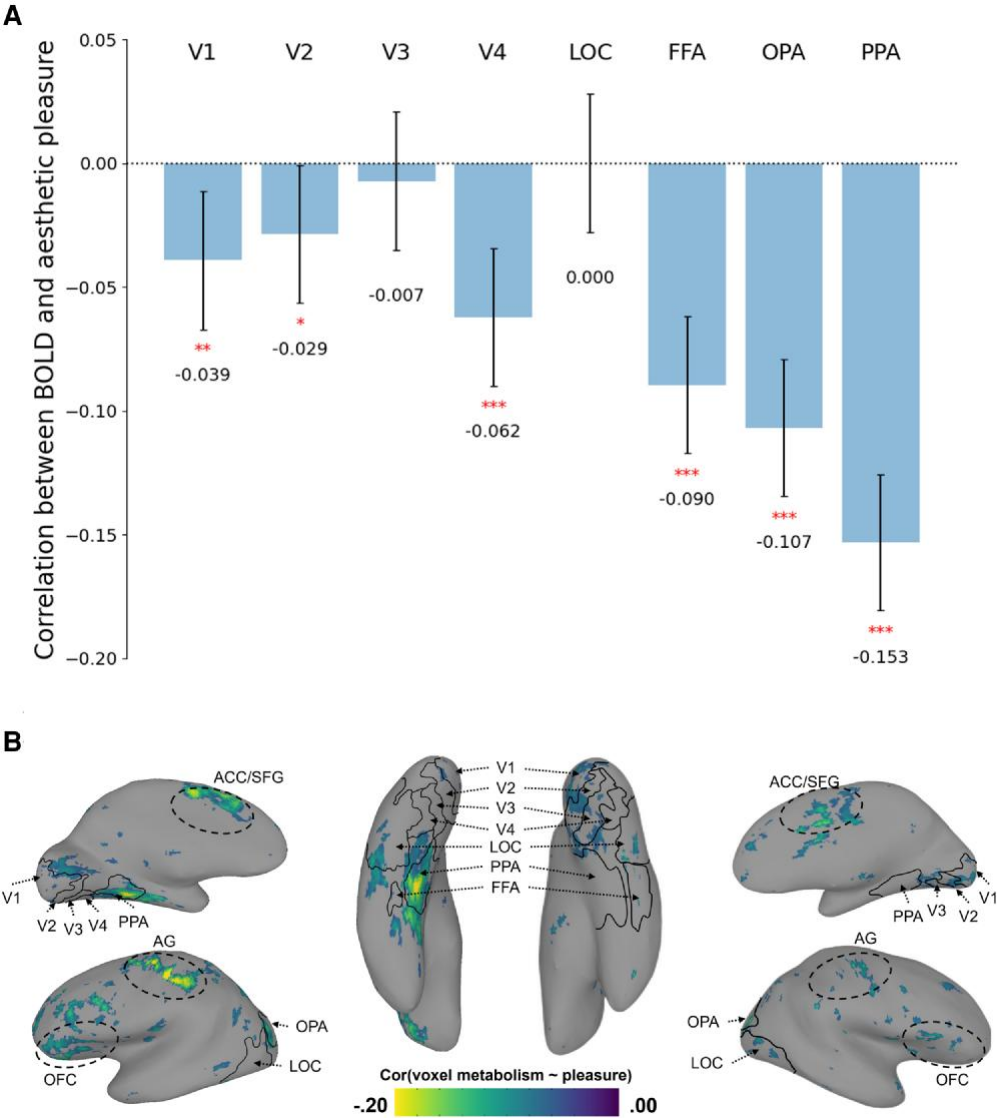
Relationship between aesthetic pleasure and metabolic costs measured by BOLD signal in fMRI. A) Negative correlations between group average BOLD activity and aesthetic pleasure. The asterisks represent statistical significance (*: 0.01 < *P* < 0.05; **: 0.001 < *P* < 0.01; ***: *P* < 0.001), and the error bars represent 95% CI. B) Inflated brain views for maps of subject 1 highlighting negative correlations between metabolic costs and aesthetic pleasure. Only negative correlations are shown here, and the threshold is *P* < 0.0001, uncorrected for multiple comparisons. AG, angular gyrus; ACC, anterior cingulate cortex; SFG, superior frontal gyrus.

We used the fMRI data of four participants viewing 4,916 images (2,900 images for one participant) from the BOLD5000 dataset to conduct item-level analysis (single-trial regression analysis) (24). We defined the metabolic costs of processing an image in a brain region as the sum of the beta values (percent signal change in a voxel in response to an experimental stimulus) of all voxels in that particular region. This parallels the definition of metabolic costs in our investigation of the deep neural network models, where we measured the sum of the activities of all model neurons. The sum values can more precisely capture the concept of metabolic costs than counting the number of active neurons. Specifically, we examined the following visual regions: V1, V2, V3, V4, the parahippocampal place area (PPA), the fusiform face area (FFA), the lateral occipital complex (LOC), and the occipital place area (OPA). These regions play critical roles in the processing of visual stimuli (visual categorization and object recognition). There was an almost universal pattern of negative correlations of the metabolic costs of each region with aesthetic preference for individual images (Fig. 3A). Specifically, V1, V2, V3, and V4 exhibited relatively weak correlations, with significant correlations observed in V1 (Spearman rho = −0.039, *P* = 0.006), V2 (Spearman rho = −0.029, *P* = 0.044), and V4 (Spearman rho = −0.062, *P* < 0.001). On the other hand, FFA (Spearman rho = −0. 033, *P* < 0.001), OPA (Spearman rho = −0.046, *P* < 0.001), and PPA (Spearman rho = −0.102, *P* < 0.001) displayed highly significant negative correlations between metabolic costs and aesthetic pleasure (no significant correlation was found in LOC, Spearman rho = 0.000, *P* = 0.999). The three regions are responsible for the recognition of scenes and faces. Thus, metabolic-related aesthetic pleasure may be mainly driven by high-level visual processing. The varying correlation strengths across regions parallel the varying contributions across layers in the VGG19 network to the variance in aesthetic pleasure, marking the different roles of different levels of representation processing. In an exploratory analysis, we tested the relationship between encoding sparseness and metabolic cost. We calculated the average sparseness of each ROI across all images using the Gini index, which better captures sparseness than commonly used measures such as the L1 norm and kurtosis (38). We then correlated these average sparseness values with the ROI-level correlations shown in Fig. 3B and found a marginal correlation, *r*(6) = 0.679, *P* = 0.064, which may indicate that ROIs with lower encoding sparseness may contribute more strongly to reductions in aesthetic pleasure through metabolic cost. Because the BOLD5000 dataset consists of images from three separate image sets, we were also interested in examining the specific correlation patterns in each stimulus set. We thus conducted separate analyses on the three sets. Across all image sets, we observed results similar to the whole BOLD5000 dataset (SI Appendix, Fig. S3; for individual-level results, see SI Appendix, Figs. S4 and S5). In all three subsets (Scenes, COCO, and ImageNet), metabolic cost was more strongly negatively correlated in high-level visual processing areas (PPA, FFA, and OPA) than in low-level processing areas (V1, V2, V2, and V4). The metabolic cost signal may be a source of the subjective sense of fluency, which has been shown to contribute to positive affect (13, 14).

Because metabolic costs at each stage of visual processing may merge into a global experience of visual discomfort (38, 39, 42), we also examined the predictive power of metabolic activity for aesthetic pleasure. Just as we did for the same analysis of the neural network activities, we fitted a multiple linear regression model to see whether the metabolic activity of the regions of interest can predict the aesthetic pleasure of the images. With 10-fold cross-validation, we found that the linear combination of the ventral stream metabolic costs significantly predicted aesthetic appreciations, with a prediction accuracy of *r* = 0.185, *P* < 0.001.

In addition to the ROI analyses, we conducted whole-brain exploratory analyses to investigate the pattern of correlations between metabolic activity and aesthetic pleasure beyond the ventral visual areas. Figure 3B shows the negative correlation maps of subject 1. It is clear that there are clusters of negative correlations in PPA and OPA, which are consistent with the results of the ROI analyses. In addition, clusters of voxels with negative correlations were also found in some parts of the prefrontal cortex (PFC), the orbitofrontal cortex (OFC), the angular gyrus (AG), the cingulate cortex, and the superior frontal gyrus (SFG). The pattern is consistent across the four participants (see SI Appendix Fig. S6 for the other three subjects).

In addition, we find clusters with positive correlations between metabolic activity and aesthetic pleasure in the medial PFC, posterior parietal cortex, precuneus, the intraparietal lobule, the tempero-parietal junction, and some parts of the anterior PFC (SI Appendix, Figs. S7 and S8). These patterns were consistent across subjects. These regions considerably overlap with the default-mode network (DMN), which is in line with previous research demonstrating a positive correlation between activations of DMN areas and aesthetic judgments (43, 44).

## Discussion

Is visual aesthetics a manifestation of an affect heuristic favoring low-energy states? Previous empirical evidence suggests that our preferences for certain visual stimuli are functional, serving as heuristics that enable us to efficiently select environments and stimuli with features indicating adaptive values (6–10). The present study proposes that aesthetic pleasure may derive from an even more fundamental heuristic: a preference for stimuli that require low metabolic costs during visual information processing.

In this study, we investigated the relationship between the aesthetic pleasure of images and the metabolic cost of their neural representations. We found that neural network models of the visual system showed an inverse correlation between aesthetic liking and metabolic cost measured as the total number of active neurons. The linear combination of layer-wise metabolic activity could predict aesthetic liking well. Critically, we only found this effect in networks trained for scene and object categorization but not in untrained models. We found the same inverse correlation between aesthetic liking and metabolic cost in the human visual system using the BOLD signal as a measure of metabolic cost. We uncovered negative correlations with ratings of image liking in brain regions ranging from low-level visual areas V1, V2, and V4, to high-level visual brain regions, such as PPA, OPA, and FFA, with stronger correlations in high- than low-level regions. Mirroring the findings in the neural network models, the involvement of such a broad range of visual regions indicates the potential roles of processing costs of both low-level visual features and scene content in the assessment of aesthetic liking.

We observed regional variations in the level of the BOLD signals. It is conceivable that cost-monitoring signals and sparse coding work in tandem to conserve energy or that sparse coding is the result of energy minimization. In an exploratory analysis, we found that the average sparseness of each region across images was positively correlated with the correlation between metabolic cost and aesthetic pleasure, *r*(6) = 0.679, *P* = 0.064. Although underpowered, this exploratory analysis trend highlights a promising direction for future research.

The inverse correlation between aesthetic pleasure and the metabolic costs of processing real-world images aligns with earlier findings in studies of visual discomfort, suggesting that pleasure-based signals may naturally incline us to favor energy-efficient states. Certain types of visual stimuli have been shown to create visual discomfort, that is, negative aesthetic liking (15–19). Visual discomfort is generated by visual stimuli that maximize neural activity and, thereby, metabolic cost, such as stripes and dot patterns (45), or high-contrast wing patterns of simulated moths (46). Consistent with our proposal, researchers investigating visual discomfort have suggested that the discomfort is possibly a homeostatic response, motivating us to seek less burdensome sensory environments and lower metabolic load (15, 16, 19). Moving beyond simple artificial stimuli, our study provides strong evidence in both models and humans with real-world images at scale, together with identifying the specific brain regions showing the inverse relationship. Future research should investigate the potential causal relationship between metabolic costs and aesthetic liking as well as explore whether aesthetic preferences genuinely reflect approach or avoidance behavioral tendencies.

The evolutionary drive toward low-energy states is also evident in the efficient and sparse coding schemes of visual processing. Given the high energy demands of the visual system (12), evolution likely favors strategies that prioritize either easily processed stimuli or structural efficiency. The sparseness of neural representations has been proposed as a fundamental principle of vision by Barlow (47, 48). Furthermore, an overcomplete set of basis functions combined with a sparsity constraint has been shown to produce visual representations resembling the receptive fields of neurons in the primary visual cortex (49). Minimizing the kurtosis of neural activity distributions produces similar results (50). The interplay between efficient neural coding and energy-conservation strategies optimizes the allocation of energy resources.

The combination of deep neural networks and fMRI analyses allows for a comprehensive multilevel investigation of the relationship between the metabolic costs of visual processing and pleasure. DCNN models offer an opportunity to examine the fundamental and straightforward feedforward calculations of visual information, free from various confounders, such as top-down effects that are difficult to disentangle in human data analyses. Factors like interest and boredom can influence the depth of processing in the visual system, obscuring the measurement of encoding metabolic costs. Recent studies have begun to adopt DCNN models to analyze the neural signal correlates of aesthetic ratings. Specifically, one major research direction explores the relationship between features of the visual stimulus encoded in neural networks and the aesthetic appreciation of that stimulus (51, 52). Another direction—where our study lies—examines the properties of the neural code. One study found that the sparsity of neural network activations, measured by the Gini index, correlates with aesthetic ratings (38). In contrast to this complex measure, our results indicate that a simpler and biologically accessible measure is also related to pleasure ratings. Beyond analyses based on DCNN models, our study includes fMRI analyses that further confirm our hypothesis, demonstrating that the effect we predicted still holds, even in the presence of potential confounders.

Our findings support the hypothesized relationship between positive-habituation and liking in Berlyne's two-factor model (20). The combination of the effects of metabolic costs and boredom explains why we definitely would not consider staring at a blank wall as the most enjoyable of all visual experiences. A stimulus needs to generate a minimum of arousal or interest in the viewer. Indeed, ratings of interest tend to be highly correlated with ratings of visual pleasure (53, 54). There seems to be a balance between sufficient stimulation of the visual system and reining in metabolic cost. This balance was recognized by Berlyne (20), who proposed an inverted U curve for the dependency of visual enjoyment on stimulus complexity, a concept that was first proposed by Wilhelm Wundt for the dependence of enjoyment as a function of stimulus intensity for simple stimuli, such as intensity of light (55). While the Wundt curve has been empirically found in a variety of applications, from neuroeconomics (56) to the enjoyment of video games (57), the processes giving rise to this inverted U shape are not yet understood. We propose that the balance between the benefit of stimulation, e.g. through information gain or direct dopaminergic activation, and metabolic cost may serve as a possible physiological mechanism of the inverted U curve.

It is important to note that the principle of minimizing metabolic cost as investigated here applies to the perceptual system, not any later deliberate cognitive processing or contemplation (37, 58, 59). Our results show a pronounced negative correlation between metabolic expense and aesthetic pleasure in high-level visual regions, such as the PPA. By contrast, previous studies have found positive correlations between fMRI activity and aesthetic pleasure in brain regions concerned with executive control and hedonic evaluation, such as the PFC, the OFC, or the DMN (43, 44, 60). The stronger engagement in those higher-level cognitive processes is also reflected in the task instructions. In our experiment, participants were asked to rate their level of pleasure, without any encouragement for further contemplation. In previous experiments, participants were required to explicitly engage in higher-level cognitive processes (“base your preference for an image on its content, rather than color, contrast, complexity, or any other low-level property of the image,” (43); “which structures you find powerful, pleasing, or profound” (43, 44)). Indeed, it has been demonstrated that viewers’ context and internal needs can influence their choice between perceptual and cognitive processing in the formation of their aesthetic appreciations (61), potentially making initially complex stimuli aesthetically pleasing with enough contemplation and cognitive elaboration (54). Thus, our results do not contradict but rather complement those previous results showing positive correlations between fMRI activity and aesthetic preference.

In addition to the potential influence of task instructions, several limitations in our study may also account for the differences between our findings and previous work. First, we used average pleasure ratings, which may bias our conclusions toward commonly liked images and obscure individual differences. However, averaging can also reduce person-specific noise and increase the power to detect any underlying relationship of interest. Second, the brief stimulus presentations, the repetitive context of the fMRI experiment, and the use of typical scene stimuli—which are likely well-understood and low in subjective complexity—may have biased participants toward fluency-based processing. If participants engaged in little deliberate processing for these reasons, our results may have limited generalizability to situations in which people engage more in deliberative processing, as we do not yet know how the brain weights the metabolic cost signal in such contexts, or whether it is suppressed. It should also be noted that metabolic cost may covary with factors such as sparsity and complexity of the stimulus. To demonstrate conclusively that the brain causally uses metabolic cost, future experiments will need to orthogonally manipulate these factors to test their effects. The negative relationship between the metabolic costs of visual processing and aesthetic pleasure aligns with current understandings of when cognitive effort adds value and when it does not. Effort, defined as the subjective intensification of mental or physical activities to intentionally achieve specific goals (62), may increase with the expenditure of metabolic resources, though they are not equivalent. Previous research indicates that cognitive effort is valued when it serves specific goals or anticipated outcomes but can be devalued when unnecessary or when effortless alternatives exist (63). While cognitive contemplation may enhance value through effort, passive viewing aligns with conditions where effort diminishes value, as individuals do not pursue volitional goals during this process. Consequently, the metabolic costs associated with processing images may be unnecessary and devalued, suggesting that the inverse correlation between metabolic costs of visual processing and pleasure reflects a default strategy for energy budget management in the absence of salient goals.

We also found that regions in the DMN exhibited positive correlations, confirming previous studies that reported a crucial role of the DMN in the generation of aesthetic experience (43, 44). The construction of aesthetic judgments may require the processing of self-relevant information in the target visual stimuli, which has been argued as a central function of the DMN. While our metabolic account of the visual system may explain the descending branch of the Wundt curve, the positive correlations found in the DMN may hint at a potential account for the ascending branch—before overly burdening the visual system, the increase of complexity may lead to an increase of self-relevance, and thus results in an increase of pleasure.

Our results are consistent with a range of prior observations and may provide a unifying framework for the understanding of visual aesthetics across different visual processing levels, considering that metabolic costs are an inescapable expense of all neural computations. First, perceptual fluency was found to contribute to aesthetic liking of visual stimuli (14). In fact, stimulus familiarity can enhance the aesthetic liking of a wide range of visual stimuli (64). However, this effect is not universal. For example, in Berlyne's study (20), repetition of simple stimuli reduced aesthetic pleasure. This finding is still compatible with the metabolic cost account: both the tedium factor and the reduction of metabolic cost may operate during repetition, but for simple stimuli, tedium predominates (20). One possible reason is that a simple stimulus is already efficiently encoded before repetition, so a further reduction in the metabolic cost of the neural representation may have little impact on aesthetic pleasure. Perceptual fluency is described as the ease of processing, that is, comparably low cognitive effort in representing and processing the stimulus. This concept is tightly related to minimizing the metabolic expense by the visual system. Computationally, such energy savings may be realized through integrative processing, which has been found to predict the perceived beauty of natural images (65). The degree of visual integration across an image is possibly a computational correlate of fluency. It refers to whether the elements of an image can be represented as a meaningful whole, thus leading to an efficient neural code. From a metabolic perspective, the larger the integration level, the more energy should be saved. Indeed, the strength of neural activity in intermediate to late layers was inversely correlated with beauty ratings (65).

In addition, exemplars of visual categories similar to the categorical prototype are generally liked the most. For instance, human faces are judged as more attractive the closer they are to the average face (66–68). Similar results hold for words (69), colors (70), and even animals and cars (71). Since categorization can be described as a compressive contraction away from the category boundary and toward the prototype (72, 73), the prototype is expected to elicit the most efficient representation and, hence, be liked the most. This prediction is consistent with the findings showing that good exemplars elicit less neural activity than bad exemplars (74–78).

Although speculative, our findings may also help explain why certain low- or mid-level visual properties, such as spatial frequency (42) and contours (6, 10, 79), influence aesthetic experiences. While existing research has observed these effects, it has not provided a clear explanation for why these features elicit affective responses. One possibility is that these properties cue survival-related benefits or dangers (6), but empirical evidence for this claim remains limited. Based on our findings, we hypothesize that these features enhance the visual system's ability to efficiently process and represent incoming information, thereby reducing metabolic demand. For instance, contours facilitate efficient scene categorization (80, 81), and manipulating contours has been shown to causally influence the affective response to scene images (79). Similarly, images with a 1/f spectrum, which aligns with the visual system's efficient coding processes, are consistently rated more positively (42).

To summarize, we present compelling evidence from both neural network models and the human visual system supporting a framework of visual aesthetics that demonstrates an inverse correlation between the metabolic cost of image representation and its aesthetic value. This relationship suggests that visual aesthetics may, in part, reflect an affect heuristic for conserving energy. Our findings may help further elucidate one of the two factors (20) proposed to explain aesthetics—namely, the positive-habituation component, or more broadly, the fluency-related aspect. Our findings also help explain and unify a broad range of empirical findings linking visual aesthetic pleasure to various perceptual and cognitive metrics. Additionally, the current findings are likely to inspire further research to determine if people also favor stimuli in other sensory domains based on the principle of conserving metabolic energy.

## Materials and methods

### Aesthetic ratings collection

Our study used an existing set of ratings collected for BOLD5000 (28). A total of 4,914 images with a resolution of 375 × 375 pixels were rated by 1,118 participants from Canada and the United States through Amazon Mechanical Turk. The participants used their personal computers and the Inquisit software (millisecond.com). They were each paid $1.50 USD for participation. In the experiment, the images were assigned randomly to groups, and 50 participants rated each image. Each participant rated 252 images in a randomized order. Participants were asked: “How much do you enjoy looking at this image?”; the response options were 1 = “not at all,” 2 = “barely enjoy,” 3 = “somewhat enjoy,” 4 = “enjoy,” and 5 = “enjoy very much.” Additionally, participants were also asked to rate the symmetry and complexity of each image (see 28 for more details).

### Model

We used a feedforward DCNN to test the hypothesized relationship between metabolic costs and aesthetics. CNNs are an ideal testbed for simulating the human visual system with high computational power and good representational similarity (29–32). Estimates of metabolic costs were calculated from CNN representations.

The VGG-19 model used in our study is a high-performance CNN visual categorization network initially trained to categorize objects and scenes using ImageNet (22). ImageNet is a large-scale database of real-world images covering a wide range of object and scene categories, contributing to the network's ability to closely match human vision (26). VGG-19 is a good computational approximation of the human visual system. When fed an image as input, the VGG-19 model generates a 1,000D vector that indicates the probabilities of the image belonging to each of the 1,000 categories. The VGG-19 architecture consists of 19 weight layers, including 16 convolution filters and 3 fully connected (FC) layers, each followed by one ReLU activation function, except for the last FC layer. The model also involves 5 max pooling steps and 1 average pooling step connecting weight layers.

To measure the metabolic costs of each layer, we first divided the VGG-19 network into 19 blocks, each block composed of one weight layer (either convolution or FC layer) and any functions performed on the layer output before the next weight layer. We recorded the outputs of each block in response to individual images. Throughout the following text, we use “layer” to refer to “block” unless otherwise specified.

The images underwent a preprocessing step to ensure consistency with the images presented to human participants during the aesthetic pleasure rating collection. This involved scaling the images to match the resolution of 375 × 375 pixels. Subsequently, the preprocessed images were fed into the neural network, and we recorded the output tensors of each layer that contained learnable weights.

To investigate whether successful categorization learning can give rise to visual aesthetic pleasure signals, we compared a pretrained VGG-19 model to 1,000 untrained models. All models were instantiated using the pytorch torchvision module. The pretrained VGG-19 model was retrieved from the torchvision.models library. The weights of each layer in the untrained models were randomly initialized with the default initialization method in PyTorch. To ensure replicability, we instantiated each untrained model with a specific random seed (from 0 to 999).

To show the generalizability of our findings, we repeated the same experiments with Resnet50 (40). This model has a superior similarity to human brain activity patterns than VGG19 (23). Therefore, replicating our results with Resnet50 is expected to further support our conclusions. Further details and results can be found in the Supporting information.

### Data analysis

To explore the relationship between the metabolic costs of a network trained on visual categorization and aesthetic judgments, we quantified each layer's metabolic cost. We measured metabolic cost as the number of activated neurons, i.e. the number of nonzero elements in each layer's output tensor. A unit's activity is considered nonzero if its value is greater than zero. By summing up the values of all the parameterized layers, we obtained the total metabolic cost of the network for each of the 4,914 images in our study. We then conducted Spearman's rank correlation to examine the relationship between the total metabolic costs of processing an image and its corresponding pleasure ratings. This operation was carried out consistently for both the pretrained model and the 1,000 randomly initialized untrained models.

To compare the metrics of the pretrained and untrained models based on the correlation scores, we performed a one-tailed nonparametric test. Specifically, we counted the number of untrained models that had a Spearman correlation smaller than that of the pretrained model. Dividing this count by the total number of untrained models gave us the level of significance as a nonparametric *P*-value.

Based on the correlation results, we conducted a regression analysis to investigate how different layers in the model contribute to the variance in image pleasure ratings. In this analysis, we considered the aesthetic pleasure of images as the outcome variable. We included the metabolic cost of each layer as regressors in the regression model, resulting in a total of 19 predictors. To ensure that our regression results were not biased towards a specific set of images, we implemented a 10-fold cross-validation procedure to compute the prediction accuracy. This procedure involved randomly and equally dividing the image set (consisting of 4,914 images), jointly with their corresponding pleasure ratings, into 10 subsets using the KFold() function from the sklearn.model_selection module in Python (shuffle = True, random_state = 0). For each unique subset, we repeated the following steps:

1. Treat the selected subset as the test data set for assessing the prediction accuracy of the regression model
2. Use the remaining nine subsets to fit the regression model
3. Fit a multiple linear regression model to the training set and generate predictions about aesthetic pleasure for the images in the test set
4. Record the predictions and coefficients of the regressors, and go back to 1 for a new subset

After completing the iteration steps for each unique subset, we assigned a predicted pleasure rating to each image (*N* = 4914) using a model that was not trained on that specific image. Subsequently, we conducted a Spearman's rank correlation test to examine the relationship between the actual and predicted pleasure ratings for each image. In addition, we computed the average weight of each regressor based on the data collected from the 10-fold cross-validation. We performed the same procedure and analyses on both the pretrained and the untrained models and then compared their correlation coefficients. To calculate the cumulative explained variance in aesthetic pleasure by layer-wise metabolic costs (Fig. 2D), we sequentially added the metabolic cost of each layer into each of the 10 regression models and recorded the total *R*-squared value at each iteration as a new regressor was included. We then calculated the average explained variance for each layer depth across the models.

### Neuroimaging data

We utilized the preprocessed neuroimaging data from the BOLD5000 dataset (24). The dataset was recorded in a slow event-related fMRI experiment. During the scans, four participants viewed the complete set of 4,916 images of the BOLD5000 image set (we discarded the data of two images for which we did not collect pleasure ratings), with one participant viewing a subset of 3,108 images. The participants were asked to rate how much they liked each image while viewing them. In total, scan sessions lasted about 15 h for each participant (9 h for one participant). Because the original 3-point rating scale may not capture the finer distinctions provided by the 5-point rating scale used in Ref. (28), we based our analyses on the latter. However, it should be noted that the results from the two rating scales are significantly correlated (Spearman's ρ = 0.410, *P* < 0.001).

Brain imaging data were collected using a 3T Siemens Verio MR scanner equipped with a 32-channel head coil at Carnegie Mellon University. Functional images were acquired using a T2*-weighted gradient recalled echo-planar imaging multiband pulse sequence. The time to repetition was set at 2 s, with an echo time of 30 ms. The imaging protocol included 69 slices with a thickness of 2 mm and no gap, aligned with the AC/PC axis. The voxel resolution was isotropic at 2 × 2 × 2 mm. For more detailed information regarding the scanning parameters, please refer to the original study (24).

The acquired raw data were preprocessed by the BOLD5000 team using GLMsingle (82). This preprocessing step involved applying a general linear model to estimate the brain-wide percent signal change in response to the experimental stimuli. The resulting GLM beta coefficients reflect the estimated percent signal change in each voxel in response to each presented image. In our study, we used them to quantify the metabolic cost in each voxel in the scanned brains during visual processing.

We used two different atlases to label regions of interest (ROIs) in our study. Regions in the early visual cortex were labeled using the probabilistic atlas developed by (83). For mid to high-level ROIs, we utilized the atlas created by (84). Based on maximum likelihood, each voxel was assigned to one of nine ROIs, including visual areas V1–4, PPA, FFA, LOC, and OPA.

We calculated the GLM beta coefficients for each ROI by aggregating the values of the voxels within it. This procedure provided us with ROI-level metabolic costs for each presented image. To ensure the reliability of the processing cost measures, we averaged the ROI-based values for repeated presentations whenever images were presented to participants multiple times. Finally, we obtained the overall image-wise metabolic cost by averaging across the four participants. We also analyzed the correlation using only the first presentation of repeated images, rather than averaging across repetitions, given the reported effects of repetition on aesthetic preference (20). The results were generally consistent with those obtained from the averaged data (see Fig. S9). In addition to testing the correlations between the metabolic costs of each ROI and the pleasure ratings, we also tested how well metabolic costs in the ROIs together can predict pleasure ratings using the same 10-fold cross-validation method that we applied in the neural network analysis. Whole-brain analyses were conducted in each subject's native space, where correlations were calculated separately between each subject's BOLD signal and the average aesthetic pleasure.

## Supplementary Material

pgaf347_Supplementary_Data

## Data Availability

Data and materials are available in the GitHub repository https://github.com/Yikai369/Metabolic_aesthetics. The code needed to reproduce the experiment and the analysis of this study is available in the GitHub repository https://github.com/Yikai369/Metabolic_aesthetics.

## Preprints

This manuscript was posted on a preprint: https://osf.io/preprints/psyarxiv/7xzvb_v2.
