# Supplementary material for: Less is more: Aesthetic liking is inversely related to metabolic expense by the visual system
Source: PNAS Nexus. 2025 Dec 2;4(12):pgaf347. doi: 10.1093/pnasnexus/pgaf347 (PMC12670382; doi:10.1093/pnasnexus/pgaf347)
Supplement: pgaf347_Supplementary_Data [file pgaf347_supplementary_data.pdf]

PNAS nexus

Supporting Information for

Less is more: Aesthetic liking is inversely related to metabolic expense by the visual system

Yikai Tang, William A. Cunningham, Dirk B. Walther

Yikai Tang
Email: yikai.tang@mail.utoronto.ca

This PDF file includes:

Figures S1 to S9
SI References

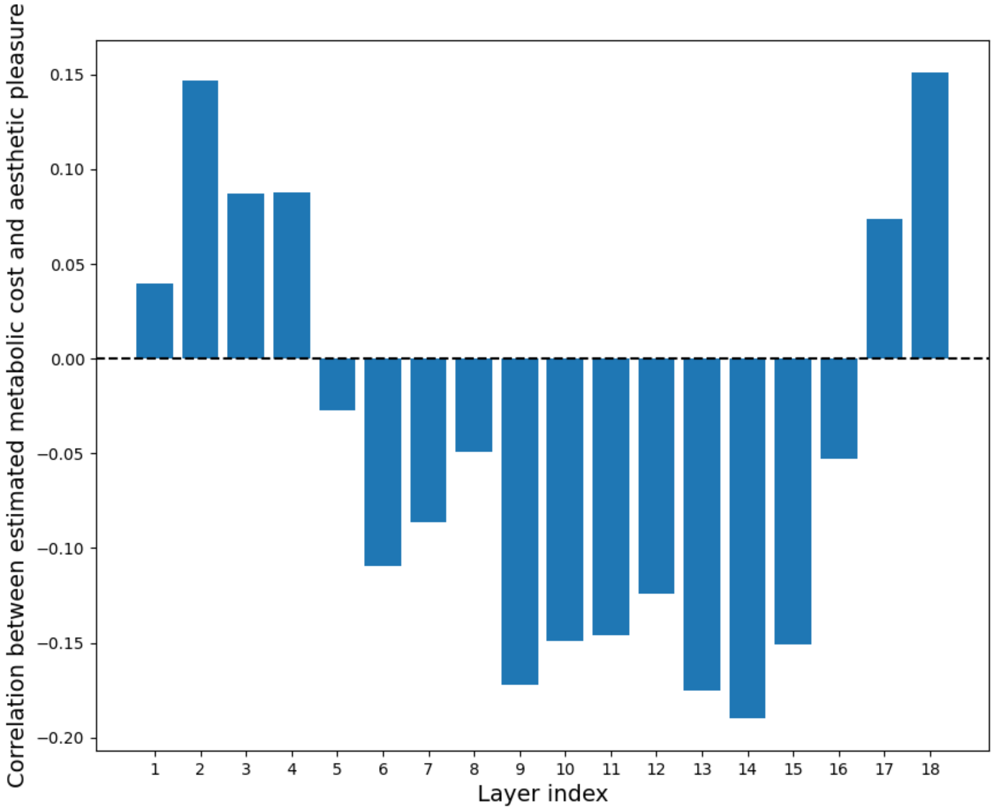

Figure S1. Correlation between aesthetic pleasure and model-derived estimates of metabolic costs for each layer in VGG19 during visual processing.

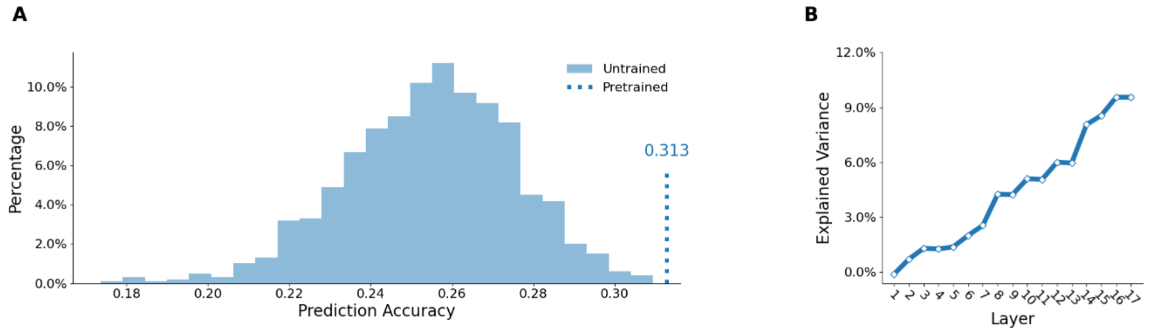

Figure S2. The linear combination of model-derived estimates of layer-wise metabolic activity in Resnet50 (1) can predict aesthetic pleasure. (A) The prediction accuracy of the regression models fitted to untrained and pre-trained Resnet50 networks' layer-wise metabolic costs, when metabolic costs were operationalized as summing up the total activations. (B) Accumulation of explained variance in aesthetic pleasure by layer-wise metabolic activity, when metabolic costs were operationalized as summing up the total activations. We evaluated the explanatory power of each layer's contribution by controlling the contributions of the layers before it.

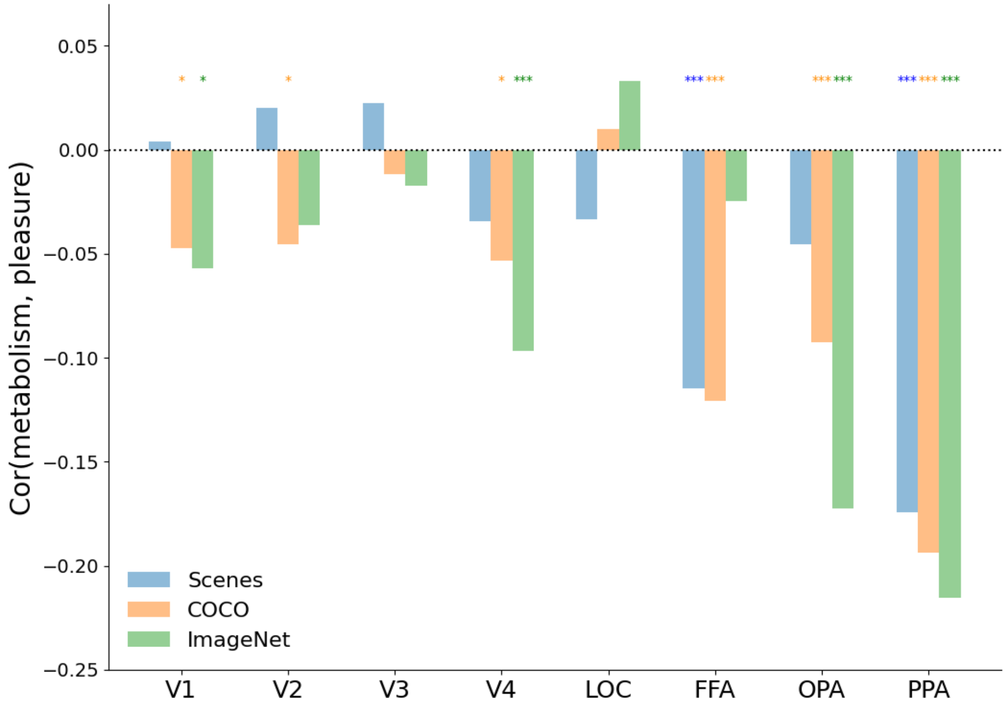

Figure S3. Correlations between BOLD activity and aesthetic appreciation for each image set, with asterisks representing statistical significance (*: .01 < p < .05; **: .001 < p < .01; ***: p < .001).

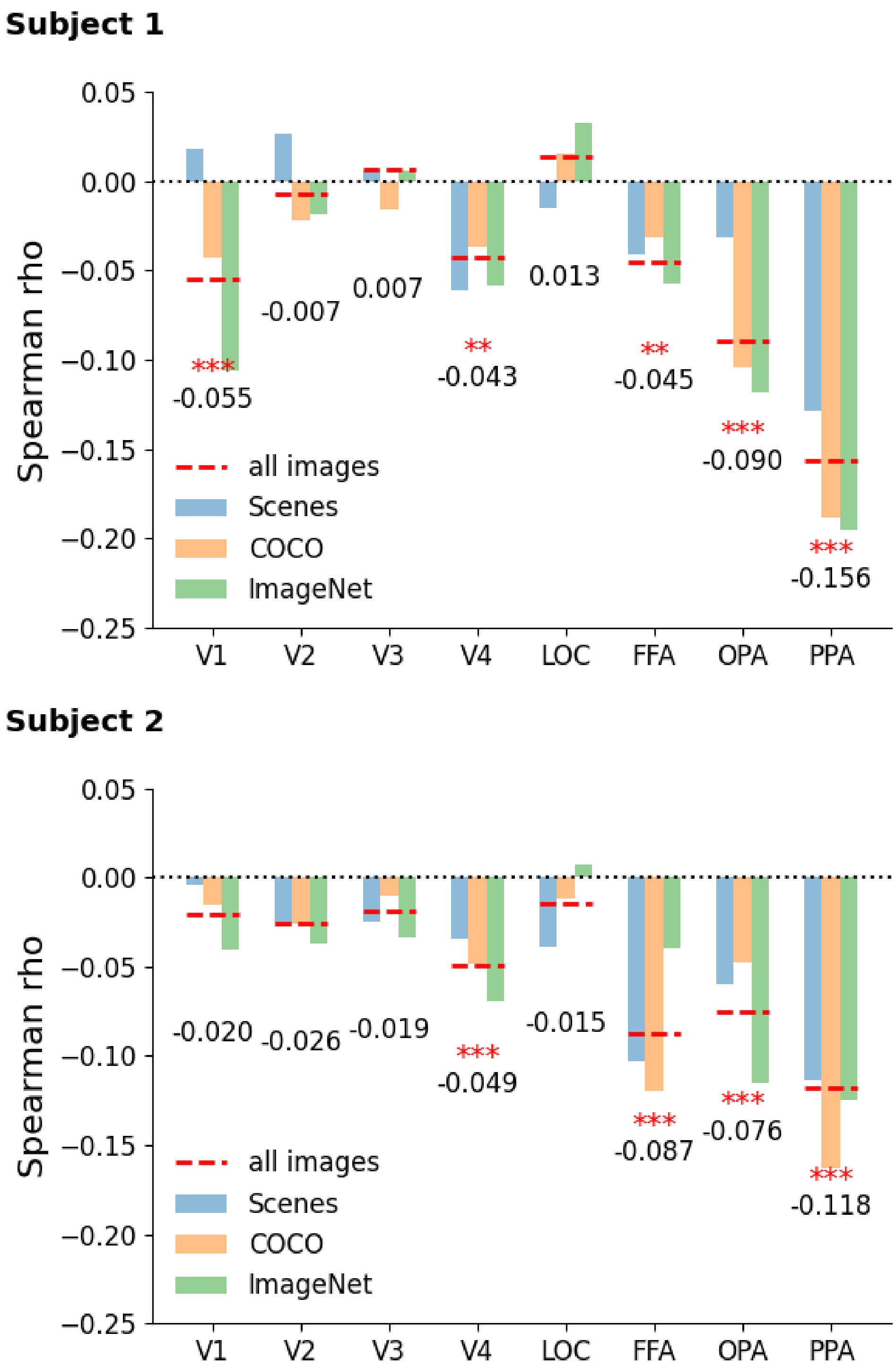

Figure S4. Correlations between BOLD activity and aesthetic pleasure for subject 1 and 2. Red dashed lines represent the results of testing all images together, with the numbers representing the Spearman rho values, and asterisks representing statistical significance (*: .01 < p < .05; **: .001 < p < .01; ***: p < .001). The colored bars represent the results of testing each subset of images separately.

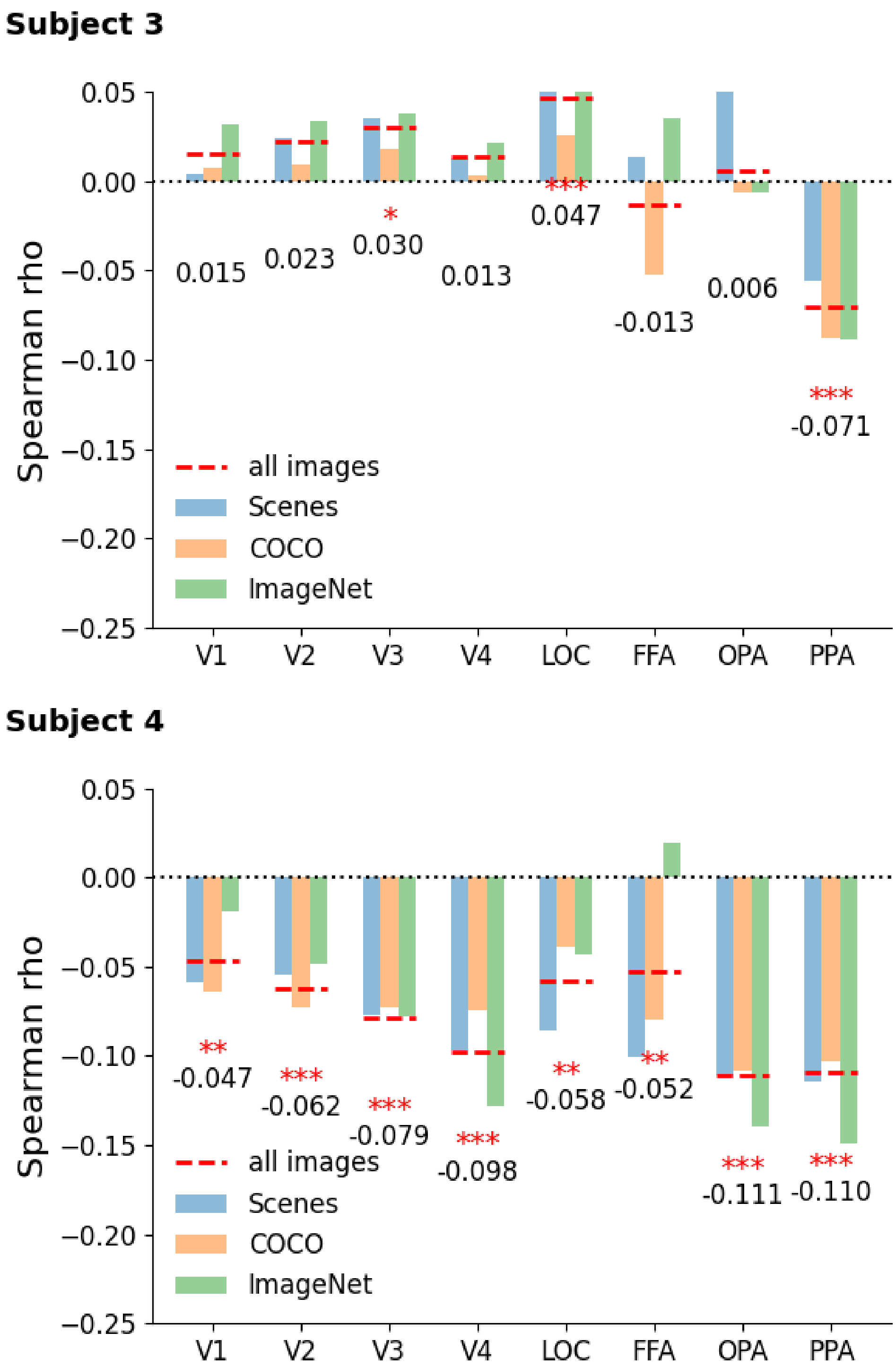

Figure S5. Correlations between BOLD activity and aesthetic pleasure for subject 3 and 4. Red dashed lines represent the results of testing all images together, with the numbers representing the Spearman rho values, and asterisks representing statistical significance (*: .01 < p < .05; **: .001 < p < .01; ***: p < .001). The colored bars represent the results of testing each subset of images separately. Subject 3 shows some positive correlation values in low-level visual areas, but most of them are not significant.

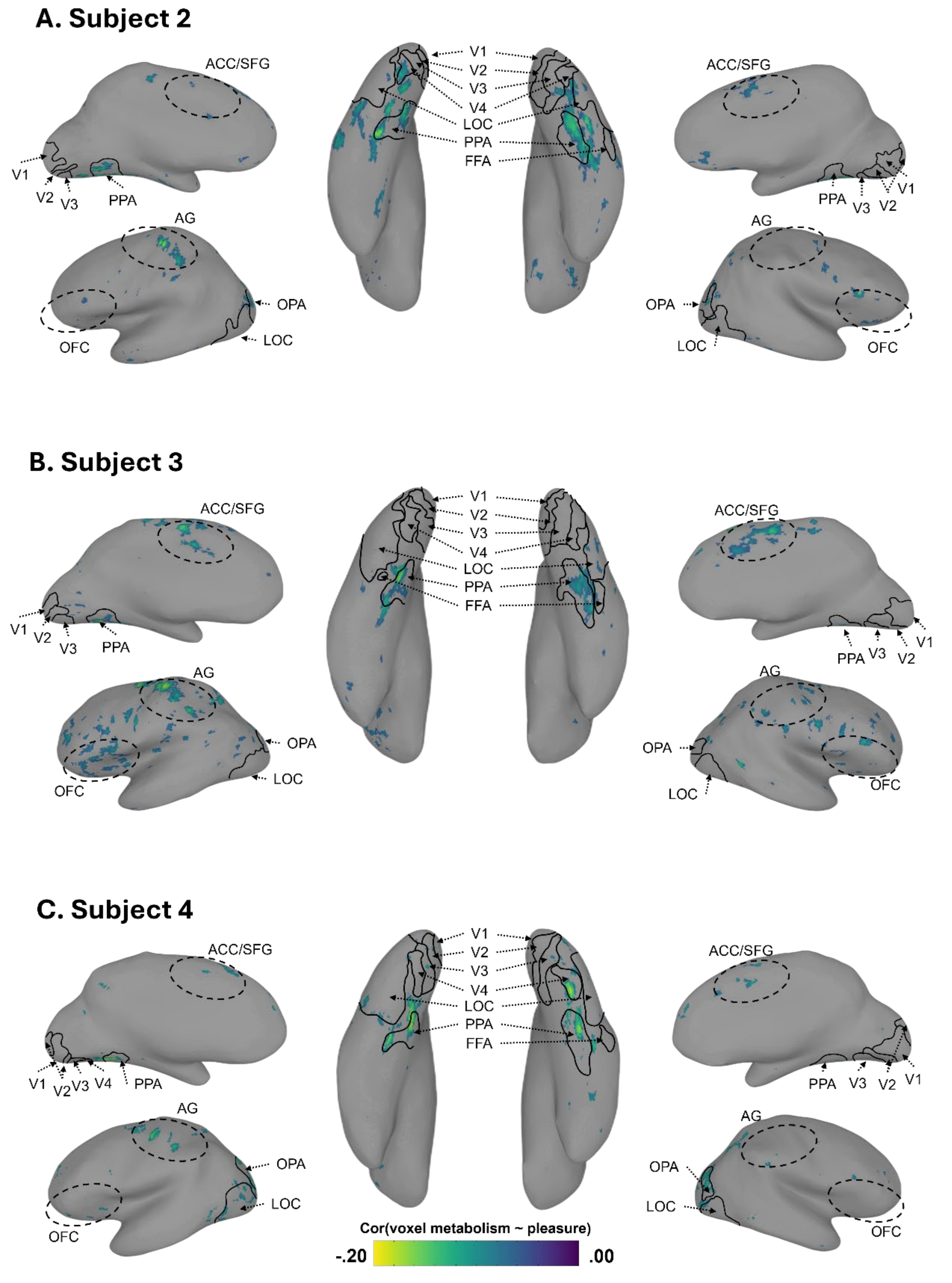

Figure S6. Inflated brain views for the maps of subject 2 (A), 3 (B), and 4 (C), highlighting negative correlations between metabolic activity and aesthetic pleasure. Only negative correlations are shown here, and the threshold is $p < .0001$, uncorrected for multiple comparisons. (AG: angular gyrus, ACC: anterior cingulate cortex, SFG: superior frontal gyrus)

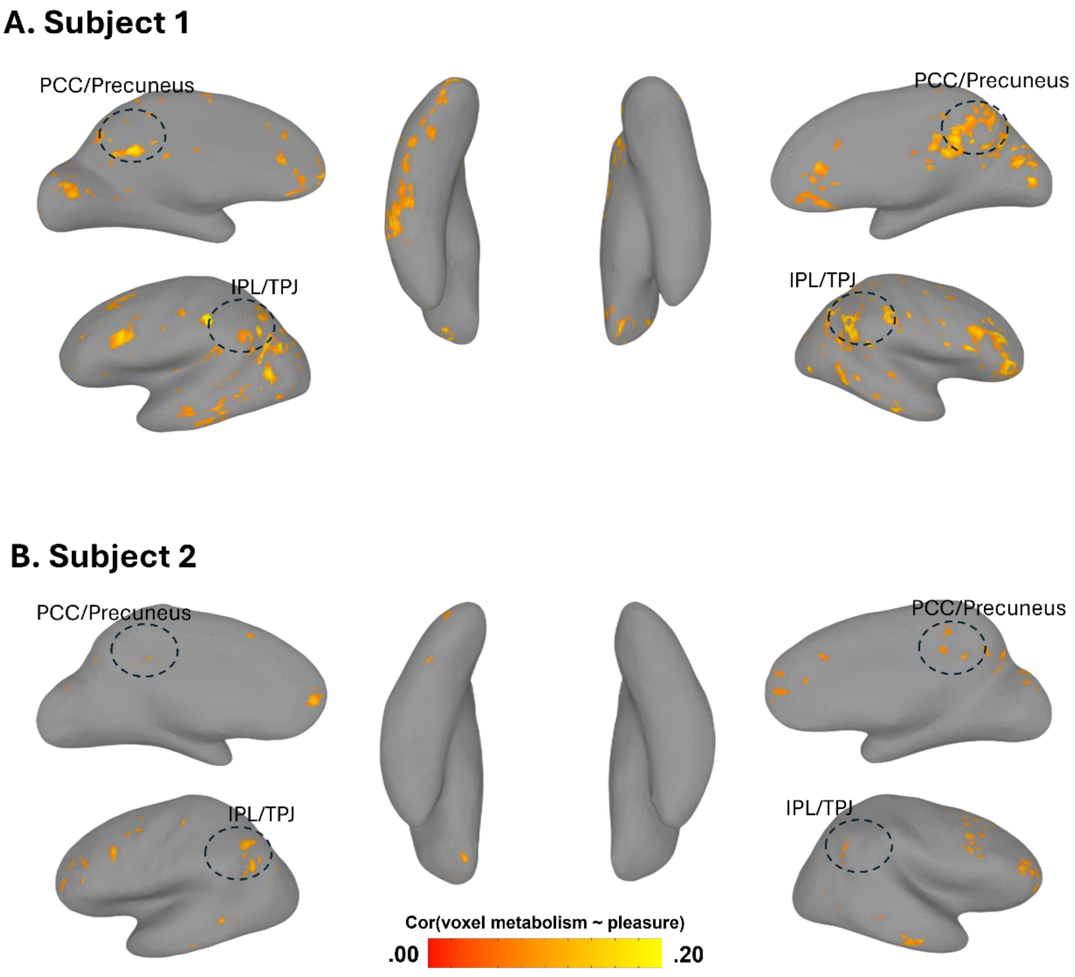

Figure S7. Inflated brain views for maps of subject 1 (A) and subject 2 (B) highlighting positive correlations between metabolic activity and aesthetic pleasure. Only positive correlations are shown here, and the threshold is $p < .0001$, uncorrected for multiple comparisons. Although voxels in Subject 1's occipital cortex show some positive correlations, the major regions in the ventral stream examined here showed inverse correlations between metabolic costs and aesthetic pleasure (Fig. S3). (IPL: inferior parietal lobule, PCC: posterior cingulate cortex, TPJ: temporoparietal junction)

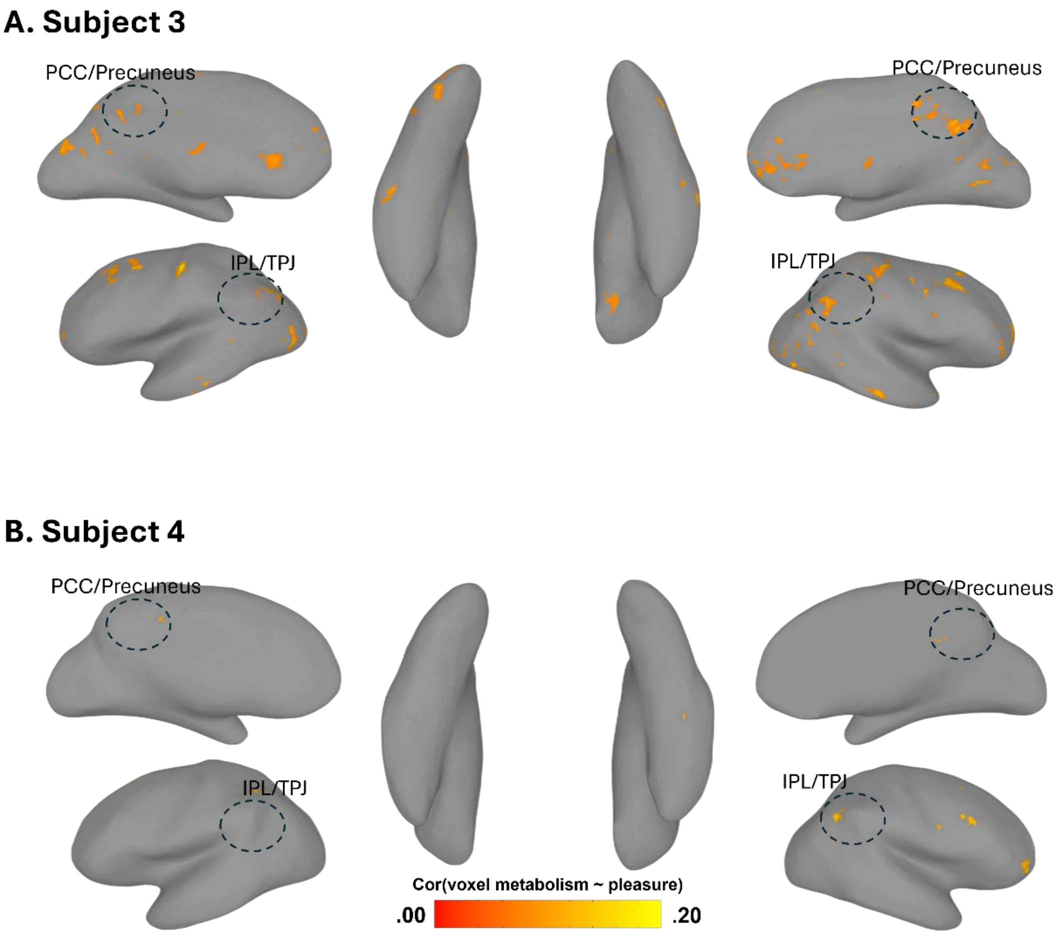

Figure S8. Inflated brain views for maps of subject 3 (A) and subject 4 (B) highlighting positive correlations between metabolic activity and aesthetic pleasure. Only positive correlations are shown here, and the threshold is $p < .0001$, uncorrected for multiple comparisons. (IPL: inferior parietal lobule, PCC: posterior cingulate cortex, TPJ: temporoparietal junction)

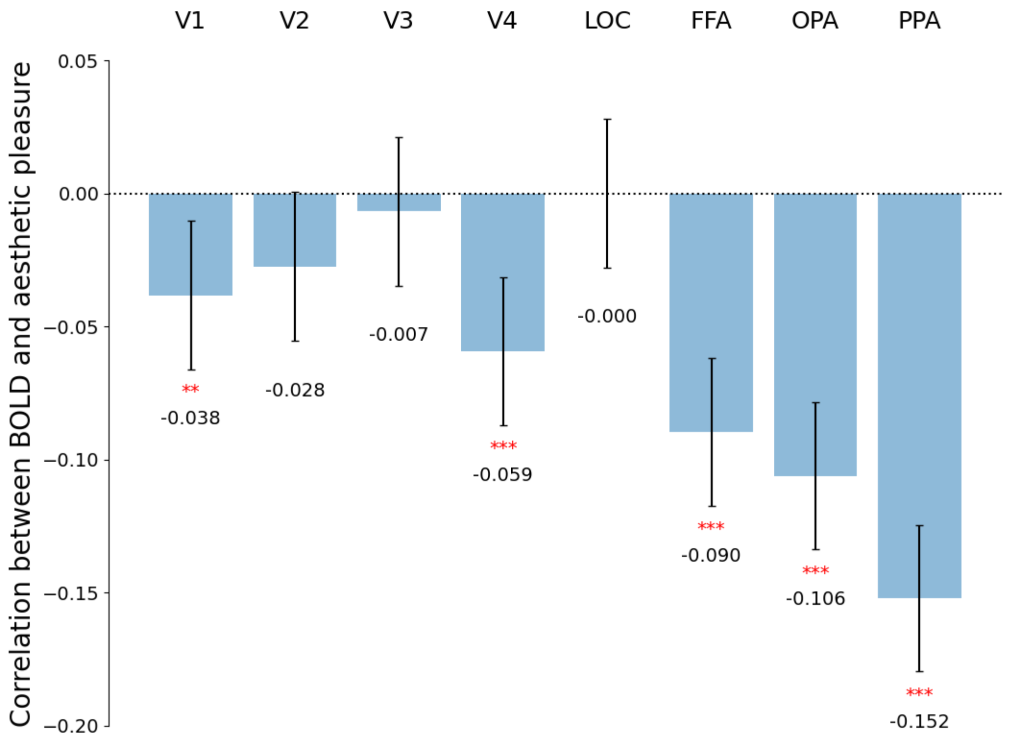

Figure S9. Negative correlations between group average BOLD activity and aesthetic pleasure. Rather than averaging across repetitions of the same images, the analysis includes only the BOLD signal from the first presentation. The asterisks represent statistical significance (*: .01 < p < .05; **: .001 < p < .01; ***: p < .001), and the error bars represent 95% confidence intervals.

SI References

1. A. Riedel, Bag of Tricks for Training Brain-Like Deep Neural Networks. *Brain-Score Workshop* (2022).
